# Evaluation of Linkage Disequilibrium, Effective Population Size and Haplotype Block Structure in Chinese Cattle

**DOI:** 10.3390/ani9030083

**Published:** 2019-03-06

**Authors:** Lei Xu, Bo Zhu, Zezhao Wang, Ling Xu, Ying Liu, Yan Chen, Lupei Zhang, Xue Gao, Huijiang Gao, Shengli Zhang, Lingyang Xu, Junya Li

**Affiliations:** 1 Institute of Animal Sciences, Chinese Academy of Agricultural Sciences, Beijing, 100193, China; xuleirock@163.com (L.X.); zhubo@caas.cn (B.Z.); wangzezhao1@163.com (Z.W.); jiujiuyake@sina.com (L.X.); yliu2333@sina.com (Y.L.); chenyan0204@163.com (Y.C.); zhanglupei@caas.cn (L.Z.); gaoxue76@126.com (X.G.); gaohj111@sina.com (H.G.); 2 National Engineering Laboratory for Animal Breeding, Key Laboratory of Animal Genetics, Breeding and Reproduction, Ministry of Agriculture, College of Animal Science and Technology, China Agricultural University, Beijing, 100193, China; zhangslcau@cau.edu.cn; 3 Institute of Animal Husbandry and Veterinary Research, Anhui Academy of Agricultural Sciences, Hefei, 230031, China

**Keywords:** linkage disequilibrium, effective population size, persistence of phase, haplotype block structure, Chinese cattle

## Abstract

**Simple Summary:**

Evaluation of the population structure and linkage disequilibrium can offer important insights to fully understand the genetic diversity and population history of cattle, which can enable us to appropriately design and implement GWAS and GS in cattle. In this study, we characterized the extent of genome-wide LD and the haplotype block structure, and estimated the persistence of phase of Chinese indigenous cattle with Illumina BovineHD BeadChip. According to our study, 58K, 87K, 95K, 52K, and 52K markers would be necessary for SCHC, NCC, SWC, SIM, and WAG, respectively, in the implementation of GWAS and GS and combining a multipopulation with high persistence of phase is feasible for the implication of genomic selection for Chinese beef cattle.

**Abstract:**

Understanding the linkage disequilibrium (LD) across the genome, haplotype structure, and persistence of phase between breeds can enable us to appropriately design and implement the genome-wide association (GWAS) and genomic selection (GS) in beef cattle. We estimated the extent of genome-wide LD, haplotype block structure, and the persistence of phase in 10 Chinese cattle population using high density BovinHD BeadChip. The overall LD measured by *r*^2^ between adjacent SNPs were 0.60, 0.67, 0.58, 0.73, and 0.71 for South Chinese cattle (SCHC), North Chinese cattle (NCC), Southwest Chinese cattle (SWC), Simmental (SIM), and Wagyu (WAG). The highest correlation (0.53) for persistence of phase across groups was observed for SCHC vs. SWC at distances of 0–50 kb, while the lowest correlation was 0.13 for SIM vs. SCHC at the same distances. In addition, the estimated current effective population sizes were 27, 14, 31, 34, and 43 for SCHC, NCC, SWC, SIM, and WAG, respectively. Our result showed that 58K, 87K, 95K, 52K, and 52K markers were required for implementation of GWAS and GS in SCHC, NCC, SWC, SIM, and WAG, respectively. Also, our findings suggested that the implication of genomic selection for multipopulation with high persistence of phase is feasible for Chinese cattle.

## 1. Introduction

High-throughput genotype technology has revolutionized genome-scale studies and offers an effective strategy to investigate population structure and genetic diversity [1]. This technology also provides new opportunities to analyze linkage disequilibrium (LD) at a genome-wide level. The power of genome-wide association study (GWAS) and predictive accuracy of genomic selection (GS) largely depend on linkage disequilibrium (LD) between quantitative trait loci (QTL) and genetic markers [2,3]. The information about LD can also provide valuable insights to explore the population history and selection, and mutations in specific genomic regions [3,4]. Extensive LD patterns can be used to characterize the selection strength in diverse cattle populations. Furthermore, as LD decays were normally found across generations, the LD level was widely utilized to estimate Ne at any particular time in the past generations [5,6]. The detection of high resolution LD pattern and characterization of haplotype block structure of the bovine genome can provide important insights into understanding economically important traits, which are under selection for different agricultural purposes [7]. Also, the extent of LD across populations depends on the maintenance of allele phase relationships between markers and QTLs [8]. Previous studies have estimated and compared the persistence of phase at various levels (across breeds, countries, or populations and generations) [9], and these findings can further be used to estimate the population history and genetic diversity [10]. Understanding of the persistence of phase between breeds are important for application of GS for small size populations as well as crossbred animals [11].

Chinese indigenous cattle have been selected for various environmental conditions and, thus, they can offer valuable resources to elucidate the genetic basis underpinning some important traits, including resistance to local diseases and parasites, fine fat deposition capabilities, adaptation to low quality feed resources, and wet-heat tolerance [12]. Many indigenous cattle normally have small population size, thus, the implementation of genomic selection in a meta-population with small population sizes will help to improve the genetic progress for these breeds. Moreover, evaluation of the extent of genome-wide LD can offer important insights to understanding the cattle genetic diversity and population history. Many studies have been carried out to quantify LD patterns using low density of markers in various indigenous cattle population from different countries and regions, such as South African [10], Spanish [13], Polish [1], Brazilian [14], Iranian [15], and Korean [16]. High density chip offers the opportunity to perform high resolution estimations of LD extension at the shortest marker distances. This genomic tool also provides a high chance to identify marker LD and construct a haplotype block map over the whole genome [17]. However, the extent of genome-wide LD and haploblock structure using BovinHD BeadChip in Chinese cattle is largely unexplored. 

In this study, we characterized the extent of genome-wide LD patterns and haplotype blocks in Chinese cattle (10 Chinese indigenous cattle populations and two imported breeds) using Illumina BovineHD BeadChip (Illumina, Inc., San Diego, CA), and the persistence of phase between breeds was estimated to evaluate the feasibility of multibreed genomic prediction for these populations.

## 2. Material and Methods

### 2.1. Animal Samples and Quality Control

All experimental procedures for animals were proposed by the Chinese Council on Animal Care and the Ministry of Agriculture of China.

The samples of Chinese indigenous cattle consisted of Mongolia cattle (MGC, *n* = 21), Yanhuang cattle (YHC, *n* = 24), Caidamu cattle (CDM, *n* = 25), Xizang cattle (XZC, *n* = 26), Pingwu cattle (PWC, *n* = 24), Liangshan cattle (LSC, *n* = 22), Zhaotong cattle (ZTC, *n* = 23), Wenshan cattle (WSC, *n* = 24), Hannan cattle (HNC, *n* = 26), and Nandan cattle (NDC, *n* = 25). Simmental cattle (SIM, *n* = 107) and Wagyu cattle (WAG, *n* = 93) were used as reference groups.

Genomic DNA was extracted from blood samples using a TIANamp Blood DNA Kit (Tiangen Biotech Company Limited, Beijing, China). The samples were genotyped using Illumina BovineHD BeadChip (Illumina, Inc., San Diego, CA, USA), which consisted of 777,962 SNPs. Genotype calling and initial data quality control (QC) were performed using Genome Studio software (Illumina Inc. San Diego, CA, USA). We divided 10 Chinese indigenous cattle populations into groups by K-means clustering implemented in R package “adegenet”. K-means is a reallocation method which uses discriminant analysis of principal components, and it allows for choosing an optimal number of clusters using a principal component analysis (PCA) procedure [18,19]. The quality control of genotype data was conducted using PLINK v1.9 [20].

Unrelated individuals were kept based on pedigree and pi-hat values. Samples with total call rates <0.90 were removed from the final dataset. Only SNPs located on autosomes were considered for subsequent analyses. SNPs with call rates <0.90, minor allele frequencies (MAF) <0.05, and those that deviated significantly from Hardy–Weinberg equilibrium (*p* < 10^−6^) were excluded.

### 2.2. Linkage Disequilibrium

The r-squared statistic can be used to estimate the extent of LD. We calculated the *r*^2^ between SNP pairs with physical distances between 0 to 1 Mb of all autosomes to estimate the extent of LD in PLINK v1.9 separately for each group. The decays of LD were analyzed for each of 2.5 kb between SNP pairs with an interval of less than 99999 SNPs and 1 Mb. To evaluate the feasibility of marker panels in GWAS and GS, the average *r*^2^ between adjacent markers was calculated according to Badke et al. [21].

### 2.3. Haplotype Blocks

Haplotype blocks are particular combinations of alleles for a genomic region in which less than 5% of comparisons among informative SNP pairs show strong evidence of historical recombination [22]. The haplotype blocks were detected across autosomes within breeds using the expectation–maximization algorithm [23] approach implemented in PLINK v1.9.

### 2.4. Persistence of Phase Across Groups

The persistence of phase between alleles on the same autosome was estimated using *r*, and the estimated parameter used was the same as the *r*-squared for SNP pairs. The persistence of phase was measured as the Pearson correlation between the average means of linkage phase in different distances. The correlations were computed for *r* between each marker pairs among all pairwise populations, and a series of genomic distance intervals were set in this analysis using bins of 2.5 kb for small genetic distance (0–10 kb), 10 kb for medium genetic distance (10–100 kb), and 100 kb for large genetic distance (100–1000 kb). 

### 2.5. Ancestral Effective Population Size

The estimated *r*^2^ was used to calculate the effective population size for past generations [24]. In general, genetic distance *c* was using physical distance approximated for estimating the Ne directly [8,25]. In this study, we calculated the Ne using the following formulations [26]:

(1) $$N_{e}=\left(\frac{1}{4c}\right)\left(\frac{1}{E\left(r^{2}\right)}-1\right),$$

(2) $$T=\frac{1}{2c},$$

where *c* is the distance in Morgan between the SNPs and assumed to be 100 Mb per Morgan. *T* represents the number of generations ago. For this part, *r*-squared was recalculated for SNP pairs with an interval less than 99999 SNPs and 20 Mb length. We inferred Ne using autosomes SNPs at distance bins of <0.025, 0.025–0.05, 0.05–0.1, 0.1–0.2, 0.2–0.4, 0.4–0.5, 0.5–0.7, 0.7–1, 1–2, 2–5, 5–10, |nd 10–50 cM. All statistical computations were conducted with R v3.0.2 [27].

## 3. Results

### 3.1. Population Structure and SNP Distribution

**Figure 1:**
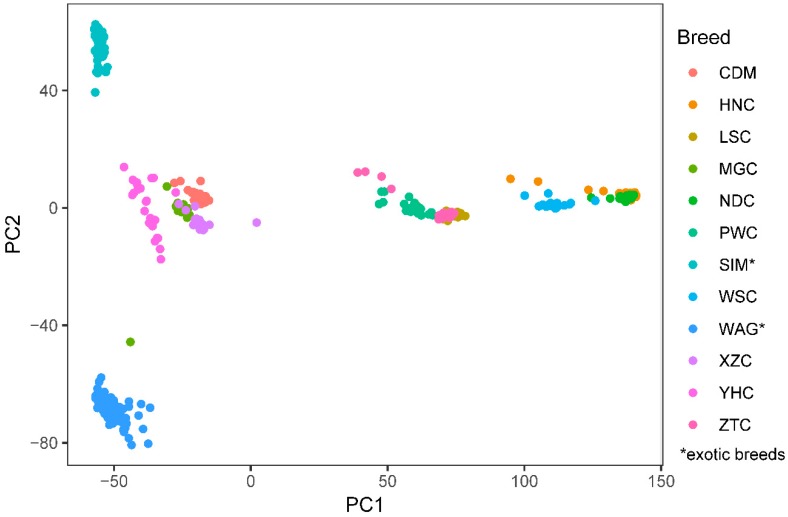
Principal component decomposition analysis of the genomic relationship in 12 cattle populations, each individual was colored by groups (PC1 and PC2 explain 12.92% and 4.97% of the proportion of variance, respectively).

All samples were genotyped using the Illumina Bovine HD BeadChip (Illumina, Inc., San Diego, CA, USA), which consisted of 777,962 SNPs with an average distance of 3.60 kb. Using this genotype data set, we divided 10 Chinese indigenous cattle populations into three groups (Figure 1), including north Chinese cattle group (NCC, contains CDM, YHC, MGC, and XZC), southwest Chinese cattle group (SWC, contains LSC, PWC, and ZTC) and south Chinese cattle group (SCHC, contains HNC, NDC and WSC), which were consistent with their geographical locations in China. Based on the cluster analysis, we found that SIM and WAG were separated from other Chinese indigenous cattle. After QC, a total of 625670 SNPs remained SNPs in NCC, while 464294 SNPs remained in SCHC. The summary of SNP distributions are presented in Supplementary Table S1. 

### 3.2. Minor Allele Frequency

**Figure 2:**
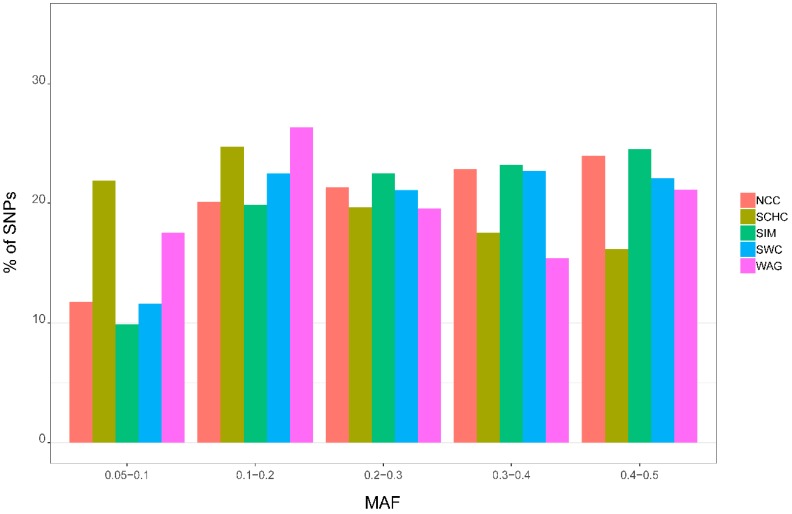
Distribution of allele frequencies in different groups. North Chinese cattle (NCC), South Chinese cattle (SCHC), Southwest Chinese cattle (SWC), Simmental (SIM), and Wagyu (WAG).

The mean MAF for autosomes SNPs were 0.24 (SCHC), 0.28 (NCC), 0.27 (SWC), 0.29 (SIM), and 0.25 (WAG). The distributions of MAFs for these groups were presented in Figure 2. The percentages of SNPs with MAFs of 0.05–0.1 were high in SCHC and WAG, while the lowest were found in SIM.

### 3.3. Extent of Linkage Disequilibrium

**Figure 3:**
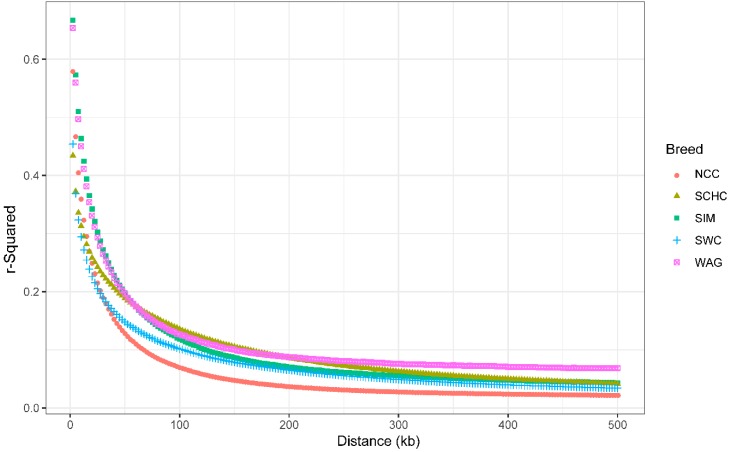
Linkage disequilibrium (LD) decay by distance across the studied groups. North Chinese cattle (NCC), South Chinese cattle (SCHC), Southwest Chinese cattle (SWC), Simmental (SIM) and Wagyu (WAG).

Across the autosomes, the overall LD (*r*^2^) between adjacent SNPs were 0.60, 0.67, 0.58, 0.73 and 0.71 for SCHC, NCC, SWC, SIM and WAG, respectively. Genome-wide average LD (*r*^2^) decreased with genetic distance for all groups (Figure 3, details in Supplementary Table S3). At genetic distances of 0–50kb, SIM had the highest LD level, followed by WAG, NCC, SWC, and SCHC. When *r*^2^ was set to 0.2, we observed that the corresponding distances between markers based on LD decay were around 45, 30, 27.5, 50, and 50 kb for SCHC, NCC, SWC, SIM, and WAG, respectively. The descriptive results of markers and LD (*r*^2^) between adjacent markers across autosomes were shown in Supplementary Table S2.

### 3.4. Haplotype Block Structure

**Figure 4:**
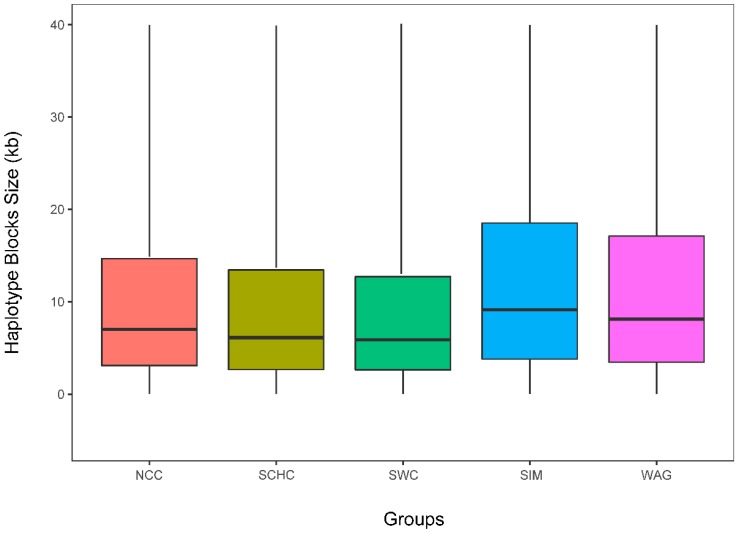
Box plots of haploblock size in different groups. North Chinese cattle (NCC), South Chinese cattle (SCHC), Southwest Chinese cattle (SWC), Simmental (SIM), and Wagyu (WAG).

The summary of genome-wide haplotype block was presented in Table 1. The total number of 61241, 88829, 86957, 72287, and 68627 haplotype blocks were detected in SCHC, NCC, SWC, SIM, and WAG respectively, and the coverage rates of cattle autosomes were 35.92%, 47.59%, 42.75%, 61.43%, and 52.25%. The distribution of haplotype block sizes is shown in Figure 4. SIM had the longest average haplotype block size, while SCHC had the shortest size. The mean number of SNPs within the haplotype blocks ranged from 4.45 (SCHC) to 7.16 (SIM). The details of shared and unique haplotype blocks for five groups were summarized in Supplementary Table S5.

### 3.5. Effective Population Size

**Figure 5:**
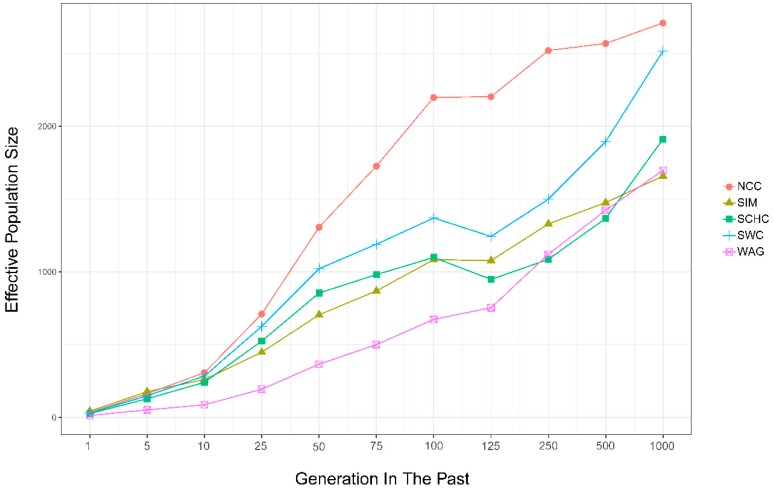
Genome-wide estimates of historical effective population size (Ne) over the past generations based on estimates of linkage disequilibrium.

As shown in Figure 5, the estimated Ne decreased over generations for studied groups (Table 2). The estimation for Ne decreased from 2710 (1000 generations ago) to 34 (recent generation) for NCC, from 1912 to 27 for SCHC, from 2517 to 31 for SWC, from1658 to 43 for SIM, and from 1696 to 14 for WAG, which reflected a decreasing trend in Ne for all breeds. In this study, NCC had the biggest estimated Ne in the recent 100 generations, while WAG had the smallest Ne.

### 3.6. Persistence of Phase

**Figure 6:**
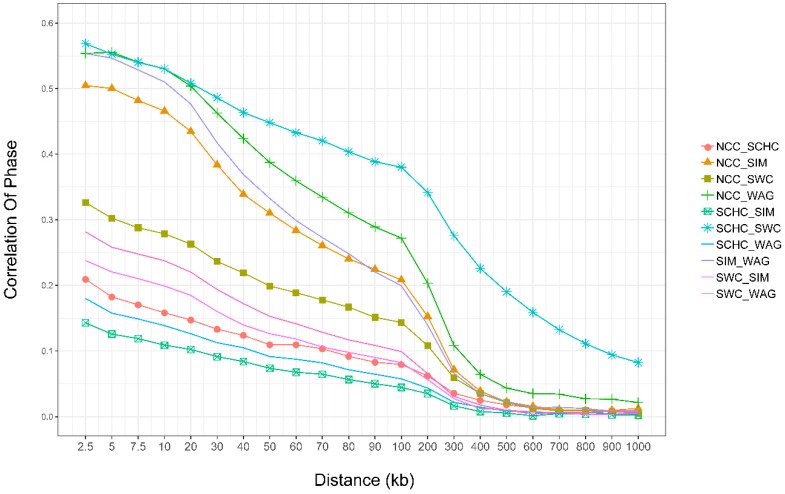
Correlation of allele phase by physical distance. North Chinese cattle (NCC), South Chinese cattle (SCHC), Southwest Chinese cattle (SWC), Simmental (SIM), and Wagyu (WAG).

In this study, the persistence of phase decreased with the distance for all groups (Figure 6, details in Supplementary Table S4). We found that the overall correlation of phase between markers in SCHC vs. SWC (RSCHC-SWC) was higher, and lower for SIM vs. SCHC. Phase correlations decreased rapidly with the increasing distances between SNPs, which was similar to the trend of average *r*^2^. RSCHC-SWC decreased from 0.57 with an average distance <2.5 kb to 0.08 for a marker distance of 900–1000 kb, while RSIM-SCHC decreased from 0.14 to 0.002. 

## 4. Discussion

### 4.1. Animals and Grouping of Chinese Indigenous Cattle

Our study is the first attempt to estimate the extent of LD and the persistence of phase among Chinese indigenous cattle using Illumina Bovine HD SNP array. Previous studies revealed that high density SNP arrays have been widely used to identify the candidate association signals for important traits [28]. Using K-means clustering [29], we drew the population structure map from the first two principal components for 10 Chinese indigenous cattle, and divided these cattle into three groups. The cluster results for these cattle are generally consistent with their geographical location. Our findings are similar to previous studies on population history [30,31,32], that Chinese indigenous cattle are mainly composed of three distinct ancestries: East Asian taurine ancestry, Eurasian taurine ancestry, and novel Chinese indicine ancestry. Small population size is a major challenge for improving the accuracy of genomic selection for indigenous cattle, thus, joint analysis of multiple populations with a certain degree of relationship is an alternative strategy for application of genome selection in different Chinese indigenous cattle with a small population size [33]. 

### 4.2. Minor Allele Frequencies (MAF) and Linkage Disequilibrium (LD)

In our study, we found Chinese cattle have higher proportion of low MAF SNPs than the imported breed (SIM and WAG), which indicates that they have a lower percentage of polymorphic loci. Our result was similar with a previous study [10]. The lower percentage of polymorphic loci among South African cattle breeds has previously been attributed to the ascertainment bias associated with the design of the BovineSNP50K BeadChip [34], as the SNP used in the design of the assay were mostly detected in European *Bos taurus* breeds, which may cause lower MAF in other breeds.

We used *r*^2^ value to estimate the extent of LD, because small sample size can cause bias for the estimates of LD when the *D*’ value was used [17]. A previous study has illustrated that the accuracy of *r*^2^ values can reach 0.85 when 55 samples were used for the calculation [17], thus, the sample size in this study was large enough (65–106 animals) for the estimation of *r*^2^ values. Moreover, we found the levels of LD were inversely related to the distance of marker pairs (Figure 3). This result is consistent with previous studies in Angus, Holstein, and other breeds [25,35,36]. The estimate of LD for SNP pairs separated by 40–50 kb was higher in SIM (0.21), WAG (0.21), and SCHC (0.20), compared to SWC (0.15) and NCC (0.14). In previous studies, the levels of LD were reported in Ethiopian cattle (0.14) [37] and Nellore cattle (0.17) [2]. The differences in the estimated LD in the current study may be ascribed to different selection and evolutionary forces during the process of breed formation [16]. As selection can change the level of LD, our result indicated that SWC and NCC may be under moderate selection compared with SCHC [38]. In our study, the highest LD values were observed in chromosome 6 (BTA6) across five groups, which may indicate the presence of QTLs with large effects that have been under intensive selection and generate high LD with nearside markers in BTA6 [39]. For instance, some QTLs at BTA6 affecting growth traits, such as birth weight [40], carcass weight [41], and ribeye area [40], and also feed intake and body weight gain [41,42], have been reported in different breeds.

Evaluation of LD levels can also be used to determine optimal marker density in GWAS and GS [43,44]. The estimated accuracy can reach up to 85% for genomic predication when the estimated *r*^2^ is greater than 0.20 [45], thus, we considered *r*^2^ greater than 0.20 as the threshold of the suggested LD level. We observed the average distance of markers pairs with *r*^2^ of 0.2, which suggested 58K, 87K, 95K, 52K, and 52K markers were required for the implementation of GS for SCHC, NCC, SWC, SIM, and WAG respectively, and that Chinese indigenous cattle need higher density markers for genomic predication compared to SIM and WAG. 

### 4.3. Haplotype Blocks

Many factors can influence the properties of haplotype blocks in cattle, including breed, marker types, and marker density of region [46]. In this study, the average haplotype block size ranged from 5.7 to 103.3 kb. Another study identified haplotype blocks in a crossbreed of Charolais and zebu, where they observed a total of 76,673 of blocks covering 1569.5 Mb (~61% of autosome genome) [47]. We observed that the number of blocks ranged from 61,241 to 88,829 across groups (Table 1). Meanwhile, the length of haplotype blocks in SIM and WAG were longer than those of the NCC, SWC, and SCHC groups. This result is probably due to the SIM and WAG having been strongly selected in recent decades compared to Chinese indigenous cattle.

We found the longest haplotype blocks at BTA5 in NCC and SWC, while the longest haplotype structures were observed at BTA2, BTA7, and BTA8 in SIM, SCHC, and WAG respectively. This result suggested the presence of beneficial variants of BTA5 propagating in NCC and SWC, which may possibly have been selected due to these traits being associated with cold tolerance and plateau adaptability. We compared the shared and unique haplotype block regions on chromosomes across groups (Supplementary Table S4), and the shared blocks covered ~14.07% of autosomes, while SIM had the largest region of unique blocks (~0.86% of autosomes) and the smallest (~0.27%) was found in NCC. This finding was probably due to SIM being derived from European cattle and they exhibit obvious genetic difference from Asian cattle (NCC, SCHC, SWC, and WAG).

### 4.4. Effective Population Size

The effective population size (Ne) can facilitate the designs of selection schemes in animal breeding [48] and the management of populations for endangered species [49]. LD pattern can be explored to comprehensively understand the population evolutionary history [50]. In this study, we computed the extent of LD at different distances between markers and estimated the ancestral effective population sizes. Ne has generally decreased since 1000 generations ago, while SCHC and SWC have increased slightly since 125–100 generations ago (Figure 5). The decrease in Ne may imply that breed formation was largely due to the post-domestication events of human migration with cattle [25]. Based on this estimation, the shrinkage of Ne depends on the number of sires and the variance of progeny number per sire [51]. We found WAG and SIM have lower Ne in the most recent 100 generations, and this could be explained by strong selection and the use of relatively few elite sires in the breeding process. 

### 4.5. Persistence of Phase

Persistence of LD was measured to assess the extent of agreement of allele phase for pairs of markers between groups [21], which can reflect the genetic relationship among them [47]. LD persistence can also be utilized to investigate the reliability of GWAS and GS across breeds. High positive values are a result of high *r*^2^ values of the same phase between two populations, whereas high negative values indicate high *r*^2^ values with a reversed phase [52]. In our study, higher correlations were found between SCHC vs. SWC, SIM vs. NCC, SIM vs. WAG, and NCC vs. WAG when the marker pair distance was 0–5 kb. The persistence of phase analysis suggested a close genetic relationship was found between SCHC and SWC, and among SIM, NCC, and WAG. This might be explained by the fact that populations from SCHC and SWC are derived from indicine, and NCC and WAG are derived from East Asian taurine [30], while SIM is from Eurasian taurine. Therefore, our result from persistence of phase analysis suggested genomic selection can be utilized for multiple small size populations with a certain degree of genetic relationship.

## 5. Conclusions

We found Chinese indigenous cattle have low LD compared to Chinese Simmental and Wagyu cattle at the genetic distance of 0–50 kb, which indicated more markers are required for the implementation of genomic selection in Chinese indigenous cattle. Our finding suggested 58K, 87K, 95K, 52K, and 52K markers were required for the implementation of genomic selection in SCHC, NCC, SWC, SIM, and WAG, respectively. Multiple population genomic selection was feasible for populations with high correlations of phase. 

## Figures and Tables

**Table 1:** Summary statistics for haploblocks across cattle groups.

Groups	NCC	SCHC	SWC	SIM	WAG
Blocks (*n*)	88,829	61,241	86,957	72,287	68,627
Total block length ^a^ (Mb)	1237.49	933.96	1111.53	1597.17	1358.55
Mean block length (kb)	13.93	15.25	12.78	22.09	19.8
SNP% in blocks	74.55%	57.88%	63.98%	86.67%	80.68%
Coverage rate of autosomes	47.59%	35.92%	42.75%	61.43%	52.25%
Mean num of SNPs in blocks	5.29	4.45	4.64	7.16	6.4
Max num of SNPs in blocks	145	122	99	225	162

^a^ Cumulative length of detected haplotype blocks.

**Table 2:** Effective population size (Ne) over the past generations based on linkage disequilibrium.

Number of Generations Ago	NCC	SCHC	SWC	SIM	WAG	Average
1	34	27	31	43	14	30
5	163	129	149	178	52	134
10	308	241	284	260	87	236
25	710	525	625	448	194	500
50	1307	854	1022	705	366	851
75	1726	981	1189	869	500	1053
100	2198	1099	1370	1083	673	1285
125	2204	948	1243	1077	753	1245
250	2521	1086	1499	1329	1121	1511
500	2569	1367	1895	1476	1422	1746
1000	2710	1912	2517	1658	1696	2099

North Chinese cattle (NCC), South Chinese cattle (SCHC), Southwest Chinese cattle (SWC), Simmental (SIM), and Wagyu (WAG).

## Supplementary Materials

The following are available online at https://www.mdpi.com/2076-2615/9/3/83/s1, Table S1: Basic information for samples. Table S2: Summary of SNPs across autosomes for five groups. Table S3: Summary of SNP pairs, average, standard deviation (SD), median linkage disequilibrium (*r*^2^) for five groups. Table S4: Summary of persistence of phase between pairwise groups for different distance. Table S5: Summary of shared and unique haplotype blocks for studied breeds.

## Abbreviations

The following abbreviations are used in this manuscript: QTL: quantitative trait loci; GWAS: genome-wide association study; GS: genomic selection; LD: linkage disequilibrium; Ne: effective population size; MAF: minor allele frequency; QC: quality control; SD: standard deviation; MGC: Inner Mongolia cattle, YHC: Yanhuang cattle, CDM: Caidamu cattle, XZC: Xizang cattle, PWC: Pingwu cattle, LSC: Liangshan cattle, ZTC: Zhaotong cattle, WSC: Wenshan cattle, HNC: Hannan cattle, NDC: Nandan cattle, SIM: Simmental cattle, WAG: Wagyu cattle, NCC: north Chinese cattle group (contains CDM, YHC, MGC, and XZC), SWC: southwest Chinese cattle group (contains LSC, PWC, and ZTC), SCHC: south Chinese cattle group (contains HNC, NDC, and WSC).
