# Supplementary material for: Evaluation of Linkage Disequilibrium, Effective Population Size and Haplotype Block Structure in Chinese Cattle
Source: Animals (Basel). 2019 Mar 6;9(3):83. doi: 10.3390/ani9030083 (PMC6466336; doi:10.3390/ani9030083)
Supplement: Supplementary file 1 [file animals-09-00083-s001.zip › Supplementary/Supplementary Table S5 Summary of shared and unique haplotype blocks for studied breeds.docx]

**Table S5.** Summary of shared and unique haplotype blocks for studied breeds.

| **Groups** | **NCC** | **SCHC** | **SWC** | **SIM** | **WAG** |
| --- | --- | --- | --- | --- | --- |
| Total Blocks Length (Mb) | 1237.39 | 933.89 | 1111.43 | 1597.02 | 1358.40 |
| Cover Rate | 47.59% | 35.92% | 42.75% | 61.43% | 52.25% |
| Unique Blocks Length (Mb) | 7.05 | 9.78 | 11.02 | 22.47 | 8.16 |
| Unique Cover Rate | 0.27% | 0.38% | 0.42% | 0.86% | 0.31% |
| Common (Mb) | 365.78 | | | | |
| Common Rate | 14.07% | | | | |

North Chinese cattle (NCC), South Chinese cattle (SCHC), Southwest Chinese cattle (SWC), Simmental (SIM), and Wagyu (WAG).
